# Estimation of average treatment effect based on a multi-index propensity score

**DOI:** 10.1186/s12874-022-01822-3

**Published:** 2022-12-28

**Authors:** Jiaqin Xu, Kecheng Wei, Ce Wang, Chen Huang, Yaxin Xue, Rui Zhang, Guoyou Qin, Yongfu Yu

**Affiliations:** 1 Department of Biostatistics, School of Public Health, Fudan University, Shanghai, China; 2 Key Laboratory of Public Health Safety of Ministry of Education, Fudan University, Shanghai, China; 3 Shanghai Institute of Infectious Disease and Biosecurity, Shanghai, China

**Keywords:** Average treatment effect, Multiply robust, Multi-index propensity score, Artificial neural network

## Abstract

**Background:**

Estimating the average effect of a treatment, exposure, or intervention on health outcomes is a primary aim of many medical studies. However, unbalanced covariates between groups can lead to confounding bias when using observational data to estimate the average treatment effect (ATE). In this study, we proposed an estimator to correct confounding bias and provide multiple protection for estimation consistency.

**Methods:**

With reference to the kernel function-based double-index propensity score (Ker.DiPS) estimator, we proposed the artificial neural network-based multi-index propensity score (ANN.MiPS) estimator. The ANN.MiPS estimator employed the artificial neural network to estimate the MiPS that combines the information from multiple candidate models for propensity score and outcome regression. A Monte Carlo simulation study was designed to evaluate the performance of the proposed ANN.MiPS estimator. Furthermore, we applied our estimator to real data to discuss its practicability.

**Results:**

The simulation study showed the bias of the ANN.MiPS estimators is very small and the standard error is similar if any one of the candidate models is correctly specified under all evaluated sample sizes, treatment rates, and covariate types. Compared to the kernel function-based estimator, the ANN.MiPS estimator usually yields smaller standard error when the correct model is incorporated in the estimator. The empirical study indicated the point estimation for ATE and its bootstrap standard error of the ANN.MiPS estimator is stable under different model specifications.

**Conclusions:**

The proposed estimator extended the combination of information from two models to multiple models and achieved multiply robust estimation for ATE. Extra efficiency was gained by our estimator compared to the kernel-based estimator. The proposed estimator provided a novel approach for estimating the causal effects in observational studies.

**Supplementary Information:**

The online version contains supplementary material available at 10.1186/s12874-022-01822-3.

## Background

Estimating the average treatment effect (ATE) is essential for assessing causal effects of treatments or interventions in biometrics, epidemiology, econometrics, and sociology. The ATE can be estimated by directly comparing mean outcomes between treated and controlled groups in randomized controlled trials [1]. However, randomized controlled trials are usually difficult to implement because of budget restrictions, ethics, and subjects’ noncompliance. Therefore, observational studies are increasingly used for estimating ATE. However, the baseline covariates are commonly unbalanced between treated and controlled groups in observational studies, and simply comparing mean outcomes may induce confounding bias [2].

Inverse probability weighting (IPW) under potential outcome framework is a popular approach for correcting confounding bias [3–5]. The IPW approach specifies a propensity score (PS) model to estimate subjects’ PS and uses the inverse of PS to balance baseline covariates between groups [6, 7]. For binary treatment, the mostly used PS model is the logistic regression. Some machine learning models, such as decision tree[8] and artificial neural network [9–12] are also used to estimate the PS. Another widely used approach is outcome regression (OR) [13]. The OR approach specifies an OR model, such as generalized linear model [14] to model the outcome as a function of the treatment and covariates to correct confounding bias directly. Some machine learning models, such as random forest [15] and artificial neural network [16] are also used as the OR model. Both IPW and OR approaches yield consistent estimation only if the corresponding model is correctly specified, but neither can be verified by the data alone.

Doubly robust approach, combining the models of PS and OR, can yield consistent estimation when any one of these two models is correctly specified (not necessarily both). Recently, a variety of doubly robust estimators for ATE have been proposed, such as augmented estimating equations estimator [17] and target maximum likelihood estimator [18]. The kernel function-based double-index propensity score (Ker.DiPS) estimator proposed by Cheng et al. [19] is one of the weighting-based doubly robust estimators. They used the Nadaraya-Watson-type kernel function to combine the information from one PS model and one OR model to obtain an integrated PS, which they named as double-index propensity score (DiPS). Using IPW approach based on the DiPS, the Ker.DiPS estimator achieved doubly robust estimation for ATE. However, the integrated PS estimated by Nadaraya-Watson-type kernel may be out of range between 0 to 1. The unreasonable PS violates the causal inference assumption and may yield uncertain estimation. Moreover, the Ker.DiPS estimator allows only two opportunities for estimation consistency.

To provide more protection on estimation consistency, we would like to develop an estimator allowing specifying multiple candidate models and can achieve estimation consistency when any one model is correctly specified. Such type of estimator is defined as multiply robust estimator [20, 21]. When combining the information from multiple candidate models to obtain the multi-index propensity score (MiPS), the Nadaraya-Watson-type kernel function may yield unstable estimation as it suffers from the “curse of dimensionality” [22–24]. With the development of scalable computing and optimization techniques [25, 26], the use of machine learning, such as artificial neural network (ANN) has been one of the most promising approaches in connection with applications related to approximation and estimation of multivariate functions [27, 28]. The ANN has the potential of overcoming the curse of dimensionality [29, 30] and has been used as a universal approximators for various functional representations [31–33]. Therefore, we replaced the kernel function with ANN to conduct nonparametric regression to estimate the MiPS. We aim to achieve multiply robust estimation for ATE using the ANN-based MiPS.

The rest of the article is organized as follows. In the Notations and assumptions section, we introduce necessary notations and causal inference assumptions. In the Some existing approaches section, we introduce some existing estimators that leads to the development of our estimator. In the Proposed multi-index propensity score section, we describe the origin and construction of the proposed estimator in detail. In the Simulation studies section, we perform simulations to evaluate the performance of the proposed estimator. A real data analysis was conducted in the Application to NHEFS data section. We make further discussion in the Discussion section and conclude the paper in the Conclusions section.

## Methods

### Notations and assumptions

Suppose that ${\mathbf{Z}}_{i}={\left({Y}_{i},{A}_{i},{\mathbf{X}}_{i}^{{\top }}\right)}^{{\top }}, i=1,\dots ,n$ be the observed data for ${i}^{\mathrm{th}}$ subject from independent and identically distributed copies of $\mathbf{Z}={\left(Y,A,{\mathbf{X}}^{{\top }}\right)}^{{\top }}$, where $Y$ is the outcome, $A$ is the binary indicator of treatment ($A=1$ if treated and $A=0$ if controlled), and $\mathbf{X}$ is the *p*-dimensional vector of pretreatment covariates. Let ${Y}^{1}$ and ${Y}^{0}$ represent the potential outcomes if a subject was assigned to treated or controlled group, respectively. The formula for average treatment effect (ATE) is

$$\Delta ={\mu }_{1}-{\mu }_{0}=E\left({Y}^{1}\right)-E\left({Y}^{0}\right).$$

Under causal inference framework, the identifiability assumptions are usually assumed, that is [6],

- *Assumption* 1. Consistency: $Y=A{Y}^{1}+(1-A){Y}^{0}$ with probability 1;
- *Assumption* 2. Ignorability: (*Y* ^1^, *Y* ^0^) ⫫ *A *| **X**, ⫫ denotes statistical independence;
- *Assumption* 3. Positivity: $0<\pi \left(\mathbf{X}\right)<1$, where $\pi \left(\mathbf{X}\right)=P\left(A=1 \right| \mathbf{X})$ denotes the propensity score.

### Some existing approaches

The IPW estimator is usually used for correcting confounding bias. The propensity score (PS) $\pi \left(\mathbf{X}\right)=P\left(A=1 \right| \mathbf{X})$ can be modeled as $\pi \left(\mathbf{X};\boldsymbol{\alpha }\right)={g}_{\pi }\left({\alpha }_{0}+{\boldsymbol{\alpha }}_{1}^{\mathrm{T}}\mathbf{X}\right)$, where ${g}_{\pi }\left(\cdot \right)$ is a specified link function, for example, the inverse of the logit function for the logistic regression, and $\boldsymbol{\alpha }={\left({\alpha }_{0},{\boldsymbol{\alpha }}_{1}^{\mathrm{T}}\right)}^{\mathrm{T}}$ are the unknown parameters and can be estimated from maximum likelihood estimation. Under causal inference assumptions, the ATE can be estimated by the IPW estimator

1 $$\begin{array}{c}{\widehat\Delta}_{IPW}=\left(\sum\limits_{i=1}^n\frac{A_i}{\pi\left({\mathbf X}_i;\widehat{\boldsymbol\alpha}\right)}\right)^{-1}\sum\limits_{i=1}^n\frac{A_i}{\pi\left({\mathbf X}_i;\widehat{\boldsymbol\alpha}\right)}Y_i-\\ \left(\sum\limits_{i=1}^n\frac{1-A_i}{1-\pi\left({\mathbf X}_i;\widehat{\boldsymbol\alpha}\right)}\right)^{-1}\sum\limits_{i=1}^n\frac{1-A_i}{1-\pi\left({\mathbf X}_i;\widehat{\boldsymbol\alpha}\right)}Y_i,\end{array}$$

where $\widehat{\boldsymbol{\alpha }}$ is the estimated value of $\boldsymbol{\alpha }$. If $\pi \left(\mathbf{X};\boldsymbol{\alpha }\right)$ is correctly specified, ${\widehat{\Delta }}_{IPW}$ is a consistent estimator of $\Delta$.

The OR estimator is another commonly used approach for correcting confounding bias. Let ${\mu }_{A}\left(\mathbf{X}\right)=E\left(Y \right| \mathbf{X},A)$ denote outcome regression (OR), where $A\in \{\mathrm{0,1}\}$. It can be modeled as ${\mu }_{A}\left(\mathbf{X};{\varvec{\beta}}\right)={g}_{\mu }\left({\beta }_{0}+{{\varvec{\beta}}}_{1}^{T}\mathbf{X}+{\beta }_{2}A\right)$, where ${g}_{\mu }(\cdot )$ is a specified link function, for example, the identity function for the linear regression, ${\varvec{\beta}}={\left({\beta }_{0},{{\varvec{\beta}}}_{1}^{{\top }},{\beta }_{2}\right)}^{{\top }}$ are the unknown parameters and can be estimated from maximum likelihood estimation. Interactions between $A$ and $\mathbf{X}$ in OR model can also be accommodated by estimating the OR separately by treated and controlled groups [19]. Under causal inference assumptions, the ATE also can be estimated by the OR estimator

2 $${\widehat{\Delta }}_{OR}=\frac{1}{n}\sum_{i=1}^{n} {\mu }_{1}\left({\mathbf{X}}_{i};\widehat{{\varvec{\beta}}}\right)-\frac{1}{n}\sum_{i=1}^{n} {\mu }_{0}\left({\mathbf{X}}_{i};\widehat{{\varvec{\beta}}}\right),$$

where $\widehat{{\varvec{\beta}}}$ is the estimated value of ${\varvec{\beta}}$. If $\mu \left(\mathbf{X},A;{\varvec{\beta}}\right)$ is correctly specified, ${\widehat{\Delta }}_{OR}$ is a consistent estimator of $\Delta$.

If the PS model for IPW estimator or the OR model for OR estimator is incorrectly specified, the estimation consistency of ${\widehat{\Delta }}_{IPW}$ or ${\widehat{\Delta }}_{OR}$ with $\Delta$ can not be guaranteed. To provide protection against model misspecification, Cheng et al. [19] considered integrating the information of PS $\pi \left(\mathbf{X};\boldsymbol{\alpha }\right)$ and OR ${\mu }_{a}\left(\mathbf{X};{\varvec{\beta}}\right)$ to construct double-index propensity score (DiPS), which is denoted by $\pi \left(\mathbf{X};{\boldsymbol{\alpha }}_{1},{{\varvec{\beta}}}_{1}\right)=E\left[A | {\boldsymbol{\alpha }}_{1}^{\mathrm{T}}\mathbf{X},{{\varvec{\beta}}}_{1}^{\mathrm{T}}\mathbf{X}\right]$. In order to estimate this conditional expectation, Cheng et al. [19] firstly got the estimated value ${\widehat{\boldsymbol{\alpha }}}_{1}$ of PS model and the estimated value ${\widehat{{\varvec{\beta}}}}_{1}$ of OR model, then used the Nadaraya-Watson kernel estimator [34] to conduct nonparametric regression of $A$ on ${\widehat{\boldsymbol{\alpha }}}_{1}^{\mathrm{T}}\mathbf{X}$ and ${\widehat{{\varvec{\beta}}}}_{1}^{\mathrm{T}}\mathbf{X}$, to get the estimated value of DiPS as

3 $$\widehat{\pi }\left(\mathbf{X};{\widehat{\boldsymbol{\alpha }}}_{1},{\widehat{{\varvec{\beta}}}}_{1}\right)=\frac{\sum_{j=1}^{n} {\mathcal{K}}_{\mathbf{H}}\left\{\left({\widehat{\mathbf{S}}}_{j}-\widehat{\mathbf{S}}\right)\right\}{A}_{j}}{\sum_{j=1}^{n} {\mathcal{K}}_{\mathbf{H}}\left\{\left({\widehat{\mathbf{S}}}_{j}-\widehat{\mathbf{S}}\right)\right\}}$$

where ${\widehat{\mathbf{S}}}_{i}=\left({\widehat{\boldsymbol{\alpha }}}_{1}^{\mathrm{T}}{\mathbf{X}}_{i},{\widehat{{\varvec{\beta}}}}_{1}^{\mathrm{T}}{\mathbf{X}}_{i}\right)$ and $\widehat{\mathbf{S}}=\left({\widehat{\boldsymbol{\alpha }}}_{1}^{\mathrm{T}}\mathbf{X},{\widehat{{\varvec{\beta}}}}_{1}^{\mathrm{T}}\mathbf{X}\right)$ are bivariate regressors, which is named double-index. ${\mathcal{K}}_{\mathbf{H}}\left(\bullet \right)$ is a kernel function with a bandwidth $\mathbf{H}$ of $2\times 2$ matrix. Using the estimated DiPS $\widehat{\pi }\left(\mathbf{X};{\widehat{\boldsymbol{\alpha }}}_{1},{\widehat{{\varvec{\beta}}}}_{1}\right)$, the ATE can be estimated by

4 $$\begin{array}{c}{\widehat\Delta}_{DiPS}=\left(\sum\limits_{i=1}^n\frac{A_i}{\widehat\pi\left({\mathbf X}_i;{\widehat{\boldsymbol\alpha}}_1,{\widehat{\beta}}_1\right)}\right)^{-1}\sum\limits_{i=1}^n\frac{A_i}{\widehat\pi\left({\mathbf X}_i;{\widehat{\boldsymbol\alpha}}_1,{\widehat{\beta}}_1\right)}Y_i-\\ \left(\sum\limits_{i=1}^n\frac{1-A_i}{1-\widehat\pi\left({\mathbf X}_i;{\widehat{\boldsymbol\alpha}}_1,{\widehat{\beta}}_1\right)}\right)^{-1}\sum\limits_{i=1}^n\frac{1-A_i}{1-\widehat\pi\left({\mathbf X}_i;{\widehat{\boldsymbol\alpha}}_1,{\widehat{\beta}}_1\right)}Y_i.\end{array}$$

Cheng et al. [19] demonstrated that ${\widehat{\Delta }}_{DiPS}$ is a doubly robust estimator: it is consistent when $\pi \left(\mathbf{X};\boldsymbol{\alpha }\right)$ is correctly specified, or ${\mu }_{A}\left(\mathbf{X};{\varvec{\beta}}\right)$ is correctly specified, but not necessarily both.

### Proposed multi-index propensity score

Although ${\widehat{\Delta }}_{DiPS}$ in (3) can achieve doubly robust estimation for ATE, the DiPS estimated by the Nadaraya-Watson kernel estimator in (2), which may make the estimated probability outside the range of 0 to1, then the above *Assumption* 3 is violated. Furthermore, ${\widehat{\Delta }}_{DiPS}$ in (3) only allows a single model for PS and a single model for OR, the estimation consistency cannot be guaranteed when both models are incorrect. To provide more protection on estimation consistency, we would like to develop an approach that allows multiple candidate models for PS and/or OR, to achieve multiple robustness: the estimator is consistent when any model for PS or any model for OR is correctly specified.

Specifically, we consider multiple candidate models for PS $\{{\pi }^{k}\left(\mathbf{X};{\boldsymbol{\alpha }}^{k}\right)={g}_{\pi }\left({\alpha }_{0}^{k}+{\boldsymbol{\alpha }}_{1}^{k\mathrm{T}}\mathbf{X}\right),k=1,\dots ,K\}$ and multiple candidate models for OR $\left\{{\mu }_{A}^{l}\left(\mathbf{X};{{\varvec{\beta}}}^{l}\right)={g}_{\mu }\left({\beta }_{1}^{l}+{{\varvec{\beta}}}_{1}^{l\mathrm{T}}\mathbf{X}+{\beta }_{2}^{l}A\right),l=1,\dots ,L\right\}$, probably with different choices or functional forms of covariates. Then we integrate the information from multiple PS models and multiple OR models to construct multi-index propensity score (MiPS), which is denoted by $\pi \left(\mathbf{X};{\boldsymbol{\alpha }}_{1}^{1},...,{\boldsymbol{\alpha }}_{1}^{K},{{\varvec{\beta}}}_{1}^{1},...,{{\varvec{\beta}}}_{1}^{L}\right)=E\left[A | {\boldsymbol{\alpha }}_{1}^{1\mathrm{T}}\mathbf{X},...{\boldsymbol{\alpha }}_{1}^{K\mathrm{T}}\mathbf{X},{{\varvec{\beta}}}_{1}^{1\mathrm{T}}\mathbf{X},...,{{\varvec{\beta}}}_{1}^{L\mathrm{T}}\mathbf{X}\right]$. In order to estimate this conditional expectation, we firstly get the estimated values ${\widehat{\boldsymbol{\alpha }}}_{1}^{1}$,…, ${\widehat{\boldsymbol{\alpha }}}_{1}^{K}$ of multiple PS models and the estimated values ${\widehat{{\varvec{\beta}}}}_{1}^{1}$,…, ${\widehat{{\varvec{\beta}}}}_{1}^{L}$ of multiple OR models, then a naive idea is to use the multivariate Nadaraya-Watson kernel estimator to conduct nonparametric regression of $A$ on ${\widehat{\boldsymbol{\alpha }}}_{1}^{1\mathrm{T}}\mathbf{X}$,…, ${\widehat{\boldsymbol{\alpha }}}_{1}^{K\mathrm{T}}\mathbf{X}$ and ${\widehat{{\varvec{\beta}}}}_{1}^{1\mathrm{T}}\mathbf{X}$,…, ${\widehat{{\varvec{\beta}}}}_{1}^{L\mathrm{T}}\mathbf{X}$ to get the estimated value of MiPS as

5 $${\widehat{\pi }}^{Ker}\left(\mathbf{X};{\widehat{\boldsymbol{\alpha }}}_{1}^{1},...,{\widehat{\boldsymbol{\alpha }}}_{1}^{K},{\widehat{{\varvec{\beta}}}}_{1}^{1},...,{\widehat{{\varvec{\beta}}}}_{1}^{L}\right)=\frac{\sum_{j=1}^{n} {\mathcal{K}}_{\mathbf{H}}\left\{\left({\widehat{\mathbf{S}}}_{j}-\widehat{\mathbf{S}}\right)\right\}{A}_{j}}{\sum_{j=1}^{n} {\mathcal{K}}_{\mathbf{H}}\left\{\left({\widehat{\mathbf{S}}}_{j}-\widehat{\mathbf{S}}\right)\right\}},$$

where ${\widehat{\mathbf{S}}}_{j}=\left({\widehat{\boldsymbol{\alpha }}}_{1}^{1\mathrm{T}}{\mathbf{X}}_{j},\dots , {\widehat{\boldsymbol{\alpha }}}_{1}^{K\mathrm{T}}{\mathbf{X}}_{j},{\widehat{{\varvec{\beta}}}}_{1}^{1\mathrm{T}}{\mathbf{X}}_{j},\dots , {\widehat{{\varvec{\beta}}}}_{1}^{L\mathrm{T}}{\mathbf{X}}_{j}\right)$ and $\widehat{\mathbf{S}}=\left({\widehat{\boldsymbol{\alpha }}}_{1}^{1\mathrm{T}}\mathbf{X},\dots , {\widehat{\boldsymbol{\alpha }}}_{1}^{K\mathrm{T}}\mathbf{X},{\widehat{{\varvec{\beta}}}}_{1}^{1\mathrm{T}}\mathbf{X},\dots , {\widehat{{\varvec{\beta}}}}_{1}^{L\mathrm{T}}\mathbf{X}\right)$ are multivariate regressors, which is named multi-index. ${\mathcal{K}}_{\mathbf{H}}\left(\bullet \right)$ is a kernel function with a bandwidth $\mathbf{H}$ of $\left(K+L\right)\times \left(K+L\right)$ matrix. Using the estimated kernel-based MiPS ${\widehat{\pi }}^{Ker}\left(\mathbf{X};{\widehat{\boldsymbol{\alpha }}}_{1}^{1},...,{\widehat{\boldsymbol{\alpha }}}_{1}^{K},{\widehat{{\varvec{\beta}}}}_{1}^{1},...,{\widehat{{\varvec{\beta}}}}_{1}^{L}\right)$, the ATE can be estimated by

6 $$\begin{array}{c}\widehat\Delta_{MiPS}^{Ker}=\left(\sum\limits_{i=1}^n\frac{A_i}{\widehat\pi^{Ker}\left({\mathbf X}_i;\widehat{\boldsymbol\alpha}_1^1,...,\widehat{\boldsymbol\alpha}_1^K,\widehat{\beta}_1^1,...,\widehat{\beta}_1^L\right)}\right)^{-1}\sum\limits_{i=1}^n\frac{A_i}{\widehat\pi^{Ker}\left({\mathbf X}_i;\widehat{\boldsymbol\alpha}_1^1,...,\widehat{\boldsymbol\alpha}_1^K,\widehat{\beta}_1^1,...,\widehat{\beta}_1^L\right)}Y_i-\\ \left(\sum\limits_{i=1}^n\frac{1-A_i}{1-\widehat\pi^{Ker}\left({\mathbf X}_i;\widehat{\boldsymbol\alpha}_1^1,...,\widehat{\boldsymbol\alpha}_1^K,\widehat{\beta}_1^1,...,\widehat{\beta}_1^L\right)}\right)^{-1}\sum\limits_{i=1}^n\frac{1-A_i}{1-\widehat\pi^{Ker}\left({\mathbf X}_i;\widehat{\boldsymbol\alpha}_1^1,...,\widehat{\boldsymbol\alpha}_1^K,\widehat{\beta}_1^1,...,\widehat{\beta}_1^L\right)}Y_i.\end{array}$$

However, if there are no additional assumptions about the regression structure, the performance of Nadaraya-Watson kernel estimator in (5) degrades as the number of regressors increases. This degradation in performance is often referred to as the “curse of dimensionality” [22–24]. Our following simulation results also show that ${\widehat{\Delta }}_{MiPS}^{Ker}$ has obvious bias when multiple candidate models are included in ${\widehat{\pi }}^{Ker}\left(\mathbf{X};{\widehat{\boldsymbol{\alpha }}}_{1}^{1},...,{\widehat{\boldsymbol{\alpha }}}_{1}^{K},{\widehat{{\varvec{\beta}}}}_{1}^{1},...,{\widehat{{\varvec{\beta}}}}_{1}^{L}\right)$, even if the correct PS and/or OR model is covered.

With the development of scalable computing and optimization techniques [25, 26], the use of machine learning has been one of the most promising approaches in connection with applications related to approximation and estimation of multivariate functions [27, 28]. Artificial neural network (ANN) is one of machine learning approaches. Benefiting from its flexible structure, the ANN becomes a universal approximator of a variety of functions [31–33]. The ANN comprises an input layer, a researcher-specified number of hidden layer(s), and an output layer. The hidden layer(s) and output layer consist of a number of neurons (also specified by researchers) with activation functions [35]. The operation of ANN includes following steps: 1) Information is input from the input layer, which passes it to the hidden layer; 2) In the hidden layer(s), the information is multiplied by the weight and a bias is added, and then passed to the next layer after transforming by the activation function; 3) The information is passed layer by layer until the last layer, where it is multiplied by the weight and then transformed by the activation function to provide the output; and 4) Calculate the error between the output and the actual value, and minimize the error by optimizing the weight parameters and bias parameters through the backpropagation algorithm [36]. In addition to having the potential of overcoming the “curse of dimensionality” [29, 30], the ANN is capable of automatically capturing complex relationships between variables [27]. It may be suited for modeling the relationship between treatment and multi-index because interactions commonly exist between indexes due to shared covariates in candidate PS and/or OR models. Therefore, we replaced the kernel function by ANN and proposed our ANN-based MiPS (ANN.MiPS) estimator.

Now we propose the ANN-based MiPS. We firstly get the estimated values ${\widehat{\boldsymbol{\alpha }}}_{1}^{1}$,…, ${\widehat{\boldsymbol{\alpha }}}_{1}^{K}$ of multiple PS models and the estimated values ${\widehat{{\varvec{\beta}}}}_{1}^{1}$,…, ${\widehat{{\varvec{\beta}}}}_{1}^{L}$ of multiple OR models, then use the ANN to conduct nonparametric regression of $A$ on multiple indexes ${\widehat{\boldsymbol{\alpha }}}_{1}^{1\mathrm{T}}\mathbf{X}$,…, ${\widehat{\boldsymbol{\alpha }}}_{1}^{K\mathrm{T}}\mathbf{X}$ and ${\widehat{{\varvec{\beta}}}}_{1}^{1\mathrm{T}}\mathbf{X}$,…, ${\widehat{{\varvec{\beta}}}}_{1}^{L\mathrm{T}}\mathbf{X}$ to get the estimated value of MiPS as ${\widehat{\pi }}^{Ann}\left(\mathbf{X};{\widehat{\boldsymbol{\alpha }}}_{1}^{1},...,{\widehat{\boldsymbol{\alpha }}}_{1}^{K},{\widehat{{\varvec{\beta}}}}_{1}^{1},...,{\widehat{{\varvec{\beta}}}}_{1}^{L}\right)$. Then the ATE can be estimated by

7 $$\begin{array}{c}\widehat\Delta_{MiPS}^{Ann}=\left(\sum\limits_{i=1}^n\frac{A_i}{\widehat\pi^{Ann}\left({\mathbf X}_i;\widehat{\boldsymbol\alpha}_1^1,...,\widehat{\boldsymbol\alpha}_1^K,\widehat{\beta}_1^1,...,\widehat{\beta}_1^L\right)}\right)^{-1}\sum\limits_{i=1}^n\frac{A_i}{\widehat\pi^{Ann}\left({\mathbf X}_i;\widehat{\boldsymbol\alpha}_1^1,...,\widehat{\boldsymbol\alpha}_1^K,\widehat{\beta}_1^1,...,\widehat{\beta}_1^L\right)}Y_i-\\ \left(\sum\limits_{i=1}^n\frac{1-A_i}{1-\widehat\pi^{Ann}\left({\mathbf X}_i;\widehat{\boldsymbol\alpha}_1^1,...,\widehat{\boldsymbol\alpha}_1^K,\widehat{\beta}_1^1,...,\widehat{\beta}_1^L\right)}\right)^{-1}\sum\limits_{i=1}^n\frac{1-A_i}{1-\widehat\pi^{Ann}\left({\mathbf X}_i;\widehat{\boldsymbol\alpha}_1^1,...,\widehat{\boldsymbol\alpha}_1^K,\widehat{\beta}_1^1,...,\widehat{\beta}_1^L\right)}Y_i.\end{array}$$

Our following simulations indicate the multiple robustness of ${\widehat{\Delta }}_{MiPS}^{Ann}$: its bias is ignorable when any model for PS or any model for OR is correctly specified.

We implemented the ANN that contains 2 hidden layers with 4 neurons in each hidden layer using AMORE package [37] for ANN.MiPS estimator. Therefore, the total number of parameters to be estimated in the ANN is $4*(K+L)+32$, including $4*(K+L)+24$ weight parameters and 8 bias parameters. The learning rate is set as 0.001 [10, 12]. The momentum is set as 0.5, the default value in the AMORE package. The hyperbolic tangent function was specified as the activation function for hidden layer. The sigmoid function was specified as the activation function for output layer to ensure the estimated ANN-based MiPS is between 0 to 1 [38]. To examine the performance stability of the estimator, we performed a sensitivity analysis using different hyperparameter selections. The simulations, real data analysis, and all statistical tests were conducted using R software (Version 4.1.0) [39]. A zip file of AMORE package and an example code for implementing the ANN.MiPS approach can be found in the attachment.

### Simulation studies

We conducted simulation studies to evaluate the performance of (i) single model-based estimators: IPW estimator in (1) and OR estimator in (2); (ii) doubly robust estimators: augmented inverse probability weighting (AIPW) [17] and target maximum likelihood estimator (TMLE) [18], which allows a single model for PS and a single model for OR; (iii) multiple models-based estimators: kernel-based estimator in (6) and ANN-based estimator in (7), which allows multiple candidate models for PS and/or OR.

**Fig. 1:**
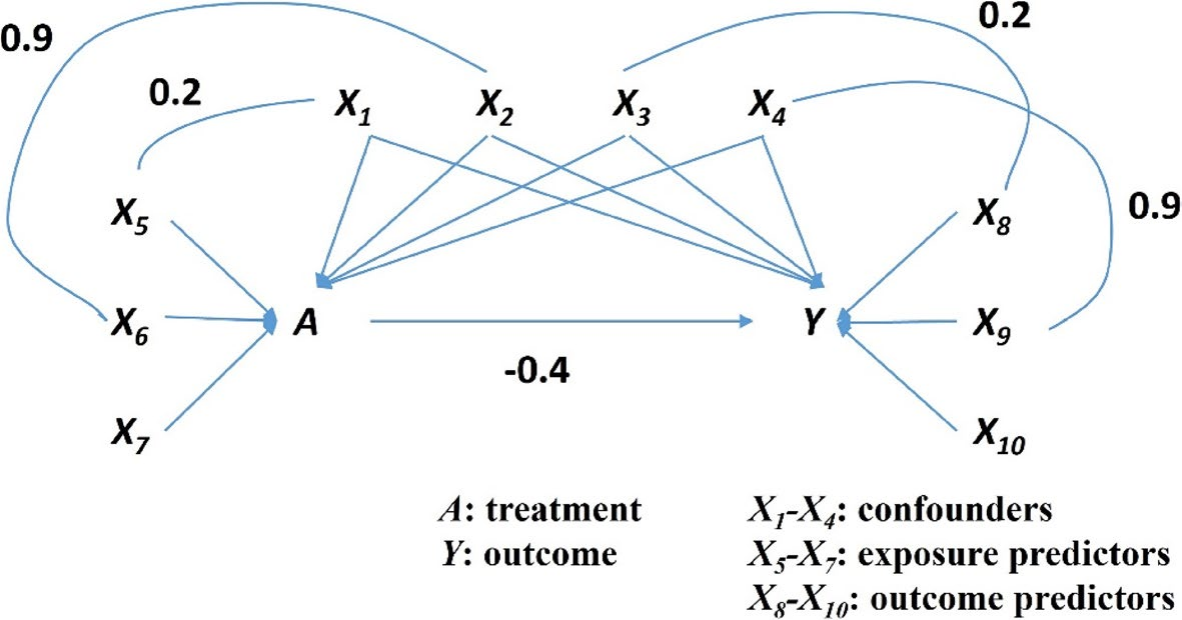
The simulation data structure in our simulation studies

Ten covariates ${X}_{1}-{X}_{10}$ were generated from standard normal distribution, and the correlation between them are shown in Fig. 1. The binary treatment indicator $A$ was generated from a Bernoulli distribution according to the following propensity score

$$\begin{array}{c}\mathrm{logit}\left[\pi\left(\mathbf X;\alpha\right)\right]=\alpha_0+0.16X_1-0.05X_2+0.12X_3-\\ 0.1X_4-0.16X_5-0.1X_6+0.15X_7\end{array}$$

${\alpha }_{0}$ was set to be 0 or -1.1 to make approximately 50% or 25% subjects entering the treatment group. The continuous outcome $Y$ was generated from

$$\begin{array}{c}Y=-3.85-0.4A-0.8X_1-0.36X_2-0.73X_3-\\ 0.2X_4+0.71X_8-0.19X_9+0.26X_{10}+\varepsilon,\end{array}$$

where $\varepsilon$ follows the standard normal distribution. The true ATE was $\Delta =E\left({Y}^{1}\right)-E\left({Y}^{0}\right)=-0.4$.

In the estimation, two estimation models were specified

$${\mathbb{A}}=\left\{\begin{array}{c}logit\left[{\pi }^{1}\left(\mathbf{X};{\boldsymbol{\alpha }}^{1}\right)\right]=\left(1,{X}_{1},{X}_{2},{X}_{3},{X}_{4},{X}_{5},{X}_{6},{X}_{7}\right){\boldsymbol{\alpha }}^{1}\\ logit\left[{\pi }^{2}\left(\mathbf{X};{\boldsymbol{\alpha }}^{2}\right)\right]=\left(1,{X}_{1}^{2},{X}_{2}^{2},{X}_{3}^{2},{X}_{4}^{2},{X}_{5}^{2},{X}_{6}^{2},{X}_{7}^{2}\right){\boldsymbol{\alpha }}^{2}\end{array}\right\}$$

for propensity score, and two estimation models were specified

$${\mathbb{B}}=\left\{\begin{array}{c}{{\mu }_{A}}^{1}\left(\mathbf{X};{{\varvec{\beta}}}^{1}\right)=\left(1,{A,X}_{1},{X}_{2},{X}_{3},{X}_{4},{X}_{8},{X}_{9},{X}_{10}\right){{\varvec{\beta}}}^{1}\\ {{\mu }_{A}}^{2}\left(\mathbf{X};{{\varvec{\beta}}}^{2}\right)=\left(1,{A,X}_{1}^{2},{X}_{2}^{2},{X}_{3}^{2},{X}_{4}^{2},{X}_{8}^{2},{X}_{9}^{2},{X}_{10}^{2}\right){{\varvec{\beta}}}^{2}\end{array}\right\}$$

for outcome regression. According to the data-generating mechanism, ${\pi }^{1}\left(\mathbf{X};{\boldsymbol{\alpha }}^{1}\right)$ and ${{\mu }_{A}}^{1}\left(\mathbf{X};{{\varvec{\beta}}}^{1}\right)$ were correct PS and correct OR models, whereas ${\pi }^{2}\left(\mathbf{X};{\boldsymbol{\alpha }}^{2}\right)$ and ${{\mu }_{A}}^{2}\left(\mathbf{X};{{\varvec{\beta}}}^{2}\right)$ were incorrect PS and incorrect OR models, due to the mis-specified functional forms of covariates. To distinguish these estimation methods, each estimator is denoted as "method-0000". Each of the four numbers, from left to right, represents if ${\pi }^{1}\left(\mathbf{X};{\boldsymbol{\alpha }}^{1}\right)$, ${\pi }^{2}\left(\mathbf{X};{\boldsymbol{\alpha }}^{2}\right)$, ${{\mu }_{A}}^{1}\left(\mathbf{X};{{\varvec{\beta}}}^{1}\right)$ or ${{\mu }_{A}}^{2}\left(\mathbf{X};{{\varvec{\beta}}}^{2}\right)$ is included in the estimator, where “1” indicates yes and “0” indicates no.

We investigated sample sizes of $n=300$ and $n=1000$ with 1000 replications in all settings. Tables 1 and 2 show the estimation results of all estimators, along with five evaluation measures including percentage of bias (BIAS, in percentage), root mean square error (RMSE), Monte Carlo standard error (MC-SE), bootstrapping standard error (BS-SE) based on 100 resamples, and coverage rate of 95% Wald confidence interval (CI-Cov). Our bootstrapping procedure resamples from the original sample set with replacement until the bootstrapping sample size reaches the original sample size. Fig. S1 shows the distribution of the estimated ATEs of Ker.MiPS and ANN.MiPS estimators. The following conclusions can be obtained. For estimation bias,

(i) If specifying one model for PS or one for OR: The IPW, Ker.MiPS, and ANN.MiPS estimators all have a small bias if the PS model is correctly specified (IPW.correct, Ker.MiPS-1000, ANN.MiPS-1000). The OR, Ker.MiPS, and ANN.MiPS estimators all have a small bias if the OR model is correctly specified (IPW.correct, Ker.MiPS-0010, ANN.MiPS-0010).
(ii) If specifying one model for PS and one model for OR: The AIPW, TMLE, Ker.MiPS and ANN.MiPS estimators all have a small bias if the PS model is correctly specified (AIPW-1010, AIPW-1001, Ker.MiPS-1010, Ker.MiPS-1001, ANN.MiPS-1010, ANN.MiPS-1001), or if the OR model is correctly specified (AIPW-1010, AIPW-0110, Ker.MiPS-1010, Ker.MiPS-0110, ANN.MiPS-1010, ANN.MiPS-0110).
(iii) If specifying multiple candidate models for PS and OR: The multiple robustness property of the ANN.MiPS estimator is well demonstrated by the ignorable bias of ANN.MiPS-1110, ANN.MiPS-1101, ANN.MiPS-1011, ANN.MiPS-0111, and ANN.MiPS-1111. On the contrary, the biases of the Ker.MiPS estimators under all model specifications are close to or larger than 5%.

**Table 1:** Estimation results under 50% treated based on 1000 replications

	*n *= 300					*n* = 1000				
Estimator	BIAS(%)	RMSE	MC-SE	BS-SE	CI-Cov(%)	BIAS(%)	RMSE	MC-SE	BS-SE	CI-Cov(%)
Single model-based estimator										
IPW.correct	-1.476	0.150	0.150	0.150	94.0	1.362	0.082	0.082	0.080	94.8
IPW.incorrect	-12.075	0.201	0.195	0.198	94.2	-10.901	0.120	0.112	0.106	92.4
IPW.ANN	-0.704	0.163	0.163	0.332	100.0	0.952	0.084	0.084	0.103	98.6
OR.correct	-0.079	0.117	0.117	0.118	93.4	1.117	0.069	0.069	0.063	92.4
OR.incorrect	-12.050	0.200	0.194	0.195	94.2	-10.752	0.120	0.112	0.106	92
OR.ANN	-3.985	0.139	0.138	0.163	97.3	-1.861	0.076	0.076	0.082	96
Doubly robust estimator										
AIPW-1010	0.113	0.119	0.119	0.120	92.8	1.128	0.069	0.069	0.064	93.4
AIPW-1001	0.856	0.154	0.154	0.157	95.4	1.328	0.083	0.083	0.081	94.4
AIPW-0110	0.022	0.119	0.119	0.121	93.0	1.135	0.069	0.069	0.064	92.4
AIPW-0101	4.900	0.203	0.197	0.199	93.8	-10.811	0.120	0.112	0.107	92
TMLE-1010	0.094	0.119	0.120	0.121	93.2	1.147	0.069	0.069	0.064	93.2
TMLE-1001	0.094	0.119	0.120	0.121	93.2	1.147	0.069	0.069	0.064	93.2
TMLE-0110	0.094	0.119	0.120	0.121	93.2	1.147	0.069	0.069	0.064	93.2
TMLE-0101	4.976	0.207	0.201	0.200	93.4	-10.771	0.120	0.113	0.107	92
Kernel regression-based MiPS estimator										
MiPS-1000	-3.698	0.152	0.151	0.196	96.2	0.959	0.083	0.083	0.161	95.8
MiPS-0100	-12.021	0.360	0.357	0.344	98.4	-8.019	0.338	0.337	0.341	97.6
MiPS-0010	-0.673	0.123	0.123	0.217	96.0	0.691	0.070	0.070	0.264	96.2
MiPS-0001	-12.457	0.316	0.313	0.364	97.4	-11.262	0.403	0.401	0.354	96.8
MiPS-1100	-5.179	0.233	0.232	0.214	96.2	4.846	0.297	0.297	0.329	98.2
MiPS-1010	-3.916	0.134	0.133	0.148	95.8	-1.373	0.075	0.075	0.135	96
MiPS-1001	-4.993	0.163	0.162	0.207	96.8	2.696	0.309	0.309	0.303	97.8
MiPS-0110	-2.545	0.147	0.146	0.168	96.4	-0.928	0.167	0.167	0.251	98
MiPS-0101	-14.182	0.262	0.256	0.311	96.6	-12.290	0.421	0.419	0.531	96.4
MiPS-0011	-4.060	0.134	0.133	0.175	96.4	1.384	0.221	0.221	0.269	98
MiPS-1110	-6.431	0.153	0.151	0.155	95.0	-4.548	0.088	0.086	0.088	91.6
MiPS-1101	-6.984	0.171	0.169	0.173	94.6	-4.906	0.125	0.123	0.152	95.6
MiPS-1011	-7.481	0.155	0.153	0.155	94.8	-4.711	0.086	0.084	0.090	94
MiPS-0111	-7.140	0.153	0.151	0.155	94.2	-4.232	0.093	0.091	0.117	95
MiPS-1111	-9.644	0.173	0.169	0.172	94.0	-7.586	0.101	0.096	0.091	91.6
Artificial neural network-based MiPS estimator										
MiPS-1000	-4.049	0.156	0.155	0.153	94.2	1.178	0.083	0.082	0.080	94.2
MiPS-0100	-11.768	0.197	0.191	0.195	94.4	-10.864	0.119	0.111	0.106	92.2
MiPS-0010	-0.927	0.119	0.119	0.122	93.4	1.156	0.069	0.069	0.064	92.2
MiPS-0001	-11.689	0.197	0.191	0.193	94.0	-10.877	0.119	0.111	0.106	92.2
MiPS-1100	-3.359	0.154	0.154	0.160	96.2	1.298	0.083	0.083	0.082	94.6
MiPS-1010	-0.033	0.123	0.123	0.132	94.8	1.056	0.070	0.070	0.066	94.2
MiPS-1001	-4.114	0.156	0.156	0.158	95.4	1.236	0.083	0.083	0.082	94.4
MiPS-0110	0.070	0.118	0.118	0.130	95.8	1.437	0.069	0.069	0.065	93
MiPS-0101	-11.762	0.198	0.192	0.197	94.8	-10.800	0.119	0.111	0.106	92
MiPS-0011	-0.663	0.119	0.119	0.123	93.6	1.250	0.069	0.069	0.064	92.4
MiPS-1110	-0.210	0.126	0.126	0.142	97.2	1.058	0.070	0.070	0.068	93.8
MiPS-1101	-3.847	0.156	0.155	0.164	95.6	1.268	0.084	0.083	0.082	94.2
MiPS-1011	0.290	0.125	0.125	0.134	95.2	1.088	0.070	0.070	0.067	94
MiPS-0111	-0.414	0.119	0.119	0.131	95.0	1.521	0.069	0.069	0.065	92.6
MiPS-1111	-0.418	0.129	0.129	0.145	96.4	1.105	0.070	0.070	0.068	94.2

The estimator which contains correct and/or incorrect models for propensity score and/or outcome regression is denoted as “method-0000”, where each digit of the four numbers, from left to right, indicates if ${\pi }^{1}\left({\varvec{X}};{\boldsymbol{\alpha }}^{1}\right)$ *,* ${\pi }^{2}\left({\varvec{X}};{\boldsymbol{\alpha }}^{2}\right)$ *,* ${{\mu }_{A}}^{1}\left({\varvec{X}};{{\varvec{\beta}}}^{1}\right)$ *or* ${{\mu }_{A}}^{2}\left({\varvec{X}};{{\varvec{\beta}}}^{2}\right)$ is included in the estimator (“1” indicates yes and “0” indicates no)

*BIAS* bias, *RMSE* root mean square error, *MC-SE* Monte Carlo standard error, *BS-SE* bootstrapping standard error, *CI-Cov* coverage rate of 95% Wald confidence interval

*AIPW* augmented inverse probability weighting, *TMLE* target maximum likelihood estimator, *IPW.ANN* artificial neural network-based inverse probability weighting estimator, *OR.ANN* artificial neural network-based outcome regression estimator, *MiPS* multi-index propensity score, *IPW* inverse probability weighting, *OR* outcome regression

**Table 2:** Estimation results under 25% treated based on 1000 replications

	*n* = 300					*n* = 1000				
Estimator	BIAS(%)	RMSE	MC-SE	BS-SE	CI-Cov(%)	BIAS(%)	RMSE	MC-SE	BS-SE	CI-Cov(%)
Single model-based estimator										
IPW.correct	-0.733	0.175	0.176	0.187	95.2	-0.111	0.098	0.098	0.095	93.0
IPW.incorrect	-12.516	0.226	0.221	0.239	96.4	-10.289	0.129	0.123	0.122	94.2
IPW.ANN	1.043	0.192	0.192	0.357	100.0	0.441	0.103	0.103	0.166	98.6
OR.correct	0.679	0.129	0.129	0.136	97.4	-0.271	0.074	0.075	0.073	94.0
OR.incorrect	-12.186	0.220	0.214	0.222	96.0	-10.228	0.130	0.123	0.121	94.0
OR.ANN	-3.761	0.164	0.164	0.164	98.4	-3.943	0.083	0.082	0.091	96.4
Doubly robust estimator										
AIPW-1010	0.432	0.137	0.137	0.143	96.4	-0.055	0.077	0.077	0.075	94.2
AIPW-1001	-0.638	0.182	0.182	0.196	96.4	-0.208	0.099	0.099	0.097	93.2
AIPW-0110	0.565	0.134	0.134	0.148	97.0	-0.304	0.075	0.075	0.074	93.6
AIPW-0101	-12.674	0.230	0.224	0.251	96.0	-10.294	0.130	0.123	0.122	93.8
TMLE-1010	-0.004	0.139	0.139	0.142	95.2	-0.029	0.077	0.077	0.075	94.2
TMLE-1001	-0.004	0.139	0.139	0.142	95.2	-0.029	0.077	0.077	0.075	94.2
TMLE-0110	-0.004	0.139	0.139	0.142	95.2	-0.029	0.077	0.077	0.075	94.2
TMLE-0101	-12.970	0.227	0.221	0.234	95.6	-10.371	0.130	0.124	0.122	93.8
Kernel regression-based MiPS estimator										
MiPS-1000	-2.459	0.179	0.179	0.226	97.8	-0.777	0.100	0.100	0.168	95.6
MiPS-0100	-6.505	0.343	0.342	0.360	97.4	-8.850	0.279	0.277	0.308	96.6
MiPS-0010	-1.988	0.140	0.140	0.226	97.8	-0.668	0.078	0.078	0.240	97
MiPS-0001	-9.204	0.328	0.326	0.347	97.0	-9.893	0.203	0.199	0.340	99.4
MiPS-1100	-4.781	0.195	0.195	0.247	96.8	-9.621	0.341	0.339	0.297	97.6
MiPS-1010	-5.620	0.166	0.165	0.176	95.2	-1.783	0.085	0.085	0.142	95.2
MiPS-1001	-3.588	0.193	0.193	0.234	96.8	-2.569	0.230	0.230	0.290	99
MiPS-0110	-3.367	0.159	0.159	0.192	97.0	1.633	0.215	0.215	0.233	96.8
MiPS-0101	-11.129	0.263	0.260	0.331	96.8	-1.934	0.467	0.468	0.480	96.6
MiPS-0011	-4.889	0.165	0.164	0.197	96.8	-2.331	0.181	0.181	0.254	98.4
MiPS-1110	-7.593	0.182	0.180	0.180	95.6	-5.415	0.099	0.097	0.101	94.6
MiPS-1101	-6.965	0.208	0.206	0.204	94.6	-5.376	0.125	0.123	0.170	94.6
MiPS-1011	-8.427	0.182	0.179	0.181	95.8	-4.716	0.101	0.099	0.104	94.4
MiPS-0111	-6.214	0.177	0.175	0.180	95.0	-5.420	0.115	0.113	0.125	95
MiPS-1111	-10.303	0.198	0.193	0.197	96.0	-7.518	0.114	0.111	0.105	92.8
Artificial neural network-based MiPS estimator										
MiPS-1000	-2.397	0.177	0.176	0.186	96.2	-0.566	0.098	0.098	0.095	93
MiPS-0100	-12.446	0.218	0.212	0.225	96.6	-10.300	0.129	0.122	0.121	94.4
MiPS-0010	0.059	0.133	0.133	0.150	98.4	-0.525	0.075	0.075	0.076	94.8
MiPS-0001	-12.252	0.216	0.211	0.221	96.0	-10.235	0.129	0.123	0.121	94
MiPS-1100	-2.543	0.184	0.184	0.200	97.6	-0.593	0.099	0.099	0.098	94
MiPS-1010	0.529	0.162	0.162	0.189	98.0	-0.395	0.083	0.084	0.087	95.2
MiPS-1001	-2.461	0.179	0.179	0.195	97.8	-0.608	0.099	0.099	0.097	93.8
MiPS-0110	0.015	0.145	0.145	0.178	99.2	-0.585	0.076	0.076	0.083	96
MiPS-0101	-12.496	0.219	0.214	0.227	96.4	-10.305	0.129	0.123	0.121	94.4
MiPS-0011	-0.014	0.134	0.134	0.155	98.4	-0.349	0.076	0.076	0.077	94.6
MiPS-1110	-1.144	0.168	0.168	0.206	98.8	-1.015	0.084	0.084	0.090	95.8
MiPS-1101	-2.636	0.188	0.188	0.206	97.2	-0.626	0.100	0.100	0.099	94
MiPS-1011	0.916	0.161	0.161	0.196	98.6	-0.446	0.084	0.084	0.089	95.4
MiPS-0111	0.227	0.143	0.143	0.183	99.4	-0.547	0.076	0.076	0.084	96.6
MiPS-1111	-0.821	0.168	0.168	0.212	98.4	-0.862	0.084	0.084	0.091	95.8

The estimator which contains correct and/or incorrect models for propensity score and/or outcome regression is denoted as “method-0000”, where each digit of the four numbers, from left to right, indicates if${\pi }^{1}\left({\varvec{X}};{\boldsymbol{\alpha }}^{1}\right)$ *,* ${\pi }^{2}\left({\varvec{X}};{\boldsymbol{\alpha }}^{2}\right)$ *,* ${{\mu }_{A}}^{1}\left({\varvec{X}};{{\varvec{\beta}}}^{1}\right)$ *or* ${{\mu }_{A}}^{2}\left({\varvec{X}};{{\varvec{\beta}}}^{2}\right)$is included in the estimator (“1” indicates yes and “0” indicates no)

*BIAS bias, RMSE root mean square error, MC-SE Monte Carlo standard error, BS-SE bootstrapping standard error, CI-Cov coverage rate of 95% Wald confidence interval*

*AIPW* augmented inverse probability weighting, *TMLE* target maximum likelihood estimator, *IPW.ANN* artificial neural network-based inverse probability weighting estimator, *OR.ANN* artificial neural network-based outcome regression estimator, *MiPS* multi-index propensity score, *IPW* inverse probability weighting, *OR* outcome regression

For estimation efficiency,

(i) If models for both PS and OR are correctly specified: The MC-SE of AIPW-1010, TMLE-1010, and ANN.MiPS-1010 estimators are all smaller than that of IPW.correct and ANN.MiPS-1000 estimators. The improved efficiency may benefit from the information of the correct OR model.
(ii) If multiple candidate models incorporate the correct PS and OR models: The MC-SE of ANN.MiPS-1110, ANN.MiPS-1011, and ANN.MiPS-1111 estimators are all close to ANN.MiPS-1010.

To evaluate the performance of the MiPS estimator when the number of specified models increases, we have considered three additional estimators: MiPS-1111-2PS, adding two additional incorrect PS models $$\left\{\begin{array}{c}logit\left[{\pi }^{3}\left(\mathbf{X};{\boldsymbol{\alpha }}^{3}\right)\right]=\left(1,{X}_{1},{X}_{2},{X}_{3}\right){\boldsymbol{\alpha }}^{3}\\ logit\left[{\pi }^{4}\left(\mathbf{X};{\boldsymbol{\alpha }}^{4}\right)\right]=\left(1,{X}_{1}^{2},{X}_{2}^{2},{X}_{3}^{2}\right){\boldsymbol{\alpha }}^{4}\end{array}\right\}$$ on the basis of the MiPS-1111; MiPS-1111-2OR, adding two additional incorrect OR models $$\left\{\begin{array}{c}{\mu }_{A}^{3}\left(\mathbf{X};{{\varvec{\beta}}}^{3}\right)=\left(1,{X}_{1},{X}_{2},{X}_{3},A\right){{\varvec{\beta}}}^{3}\\ {\mu }_{A}^{4}\left(\mathbf{X};{{\varvec{\beta}}}^{4}\right)=\left(1,{X}_{1}^{2},{X}_{2}^{2},{X}_{3}^{2},A\right){{\varvec{\beta}}}^{4}\end{array}\right\}$$ on the basis of the MiPS-1111; MiPS-1111-2PS-2OR, adding two additional incorrect PS models ${\pi }^{3}\left(\mathbf{X};{\boldsymbol{\alpha }}^{3}\right)$ and ${\pi }^{4}\left(\mathbf{X};{\boldsymbol{\alpha }}^{4}\right)$ and two additional incorrect OR models ${\mu }_{A}^{3}\left(\mathbf{X};{{\varvec{\beta}}}^{3}\right)$ and ${\mu }_{A}^{4}\left(\mathbf{X};{{\varvec{\beta}}}^{4}\right)$ on the basis of the MiPS-1111. Table 3 shows the estimation results. The following conclusions can be obtained.

(i) The estimation bias of ANN.MiPS-1111-2PS, ANN.MiPS-1111-2OR, and ANN.MiPS-1111-2PS2OR estimators is still ignorable. The estimation efficiency of these estimators is hardly degraded compared to ANN.MiPS-1010 estimator.
(ii) The estimation bias of Ker.MiPS-1111-2PS, Ker.MiPS-1111-2OR, and Ker-1111-2PS2OR estimators is close to or larger than 10%. The MC-SE of these estimators is obviously larger than that of Ker.MiPS-1010 estimator.

**Table 3:** Estimation results for multi-index propensity score estimator incorporating extra incorrect models based on 1000 replications

	*n* = 300					*n* = 1000				
Estimator	BIAS(%)	RMSE	MC-SE	BS-SE	CI-Cov(%)	BIAS(%)	RMSE	MC-SE	BS-SE	CI-Cov(%)
Under 25% treated										
Kernel regression-based MiPS estimator										
MiPS-1111-2PS	-11.969	0.207	0.202	0.212	96.4	-9.355	0.123	0.118	0.115	93.4
MiPS-1111-2OR	-11.959	0.208	0.203	0.212	96.6	-9.304	0.123	0.117	0.115	94.2
MiPS-1111-2PS2OR	-12.417	0.213	0.207	0.217	96.4	-9.966	0.127	0.121	0.119	93.8
Artificial neural network-based MiPS estimator										
MiPS-1111-2PS	-0.391	0.170	0.170	0.217	98.8	-0.842	0.084	0.084	0.092	96
MiPS-1111-2OR	-0.262	0.169	0.169	0.218	98.8	-0.645	0.085	0.085	0.092	96
MiPS-1111-2PS2OR	-0.687	0.173	0.174	0.222	99.2	-0.827	0.084	0.084	0.093	96
Under 50% treated										
Kernel regression-based MiPS estimator										
MiPS-1111-2PS	-10.967	0.189	0.184	0.186	94.4	-9.795	0.113	0.106	0.101	91.8
MiPS-1111-2OR	-10.971	0.189	0.184	0.186	94.8	-9.907	0.113	0.106	0.101	92.6
MiPS-1111-2PS2OR	-11.444	0.194	0.189	0.191	94.4	-10.583	0.118	0.110	0.104	92.6
Artificial neural network-based MiPS estimator										
MiPS-1111-2PS	-0.376	0.128	0.129	0.146	97.0	1.034	0.070	0.070	0.068	94
MiPS-1111-2OR	-0.781	0.127	0.127	0.146	97.8	0.993	0.071	0.071	0.069	94.2
MiPS-1111-2PS2OR	-0.480	0.129	0.129	0.147	97.2	0.988	0.070	0.070	0.069	94.4

MiPS-1111-2PS indicates the estimator with two additional incorrect propensity score models on the basis of MiPS-1111 estimator

MiPS-1111-2OR indicates the estimator with two additional incorrect outcome regression models on the basis of MiPS-1111 estimator

MiPS-1111-2PS2OR indicates the estimator with two additional two incorrect propensity score and 2 incorrect outcome regression models on the basis of MiPS-1111 estimator

*BIAS* bias, *RMSE* root mean square error, *MC-SE* Monte Carlo standard error, *BS-SE* bootstrapping standard error, *CI-Cov* coverage rate of 95% Wald confidence interval, *MiPS* multi-index propensity score

We also evaluated the performance of ANN.MiPS estimator under the simulation scenario with both continuous and discrete covariates. The simulation setting was described in Supplementary Document. Similar conclusions can be obtained as the above scenario with all continuous covariates (Table S1, S2). The sensitivity analysis of hyperparameters selection in ANN revealed the performance stability of ANN.MiPS estimator (Table S3).

### Application to NHEFS data

To illustrate our proposed method, we analyzed a subset of real data from the National Health and Nutrition Examination Survey Data | Epidemiologic Follow-up Study (NHEFS) (wwwn.cdc.gov/nchs/nhanes/nhefs/). The dataset consists of 1,507 participants aged 25–74 who smoked at the first survey and were followed for approximately 10 years. The empirical study aimed to estimate the ATE of smoking cessation (coded as quitting and non-quitting, with non-quitting as the reference group) on weight gain. Participants were categorized as treated if they quit smoking during follow-up, otherwise controlled. Weight gain for each individual was measured as weight at the end of follow-up minus weight at baseline survey (in kilograms). During the 10-year follow-up, 379 (25.15%) participants quit smoking. The average weight gain was greater for those who quit smoking with an unadjusted difference of 2.4 kg.

Table 4 summarized the baseline characteristics, including age, gender, race, baseline weight, active life level, education level, exercise, smoking intensity, smoking years, and ever use of weight loss medication between the smoking quitters and non-quitters. As shown in the table, the distribution of age, gender, race, education level, smoking intensity, and smoking years was different between quitters and non-quitters. When estimating the ATE of smoking cessation on weight gain, these factors should be adjusted for if they are confounders.

**Table 4:** The NHEFS data analysis: baseline characteristics between non-quitters and quitters

Characteristic	Non-quitters	Quitters	*P*-value
	*N* = 1128, 74.85%	*N* = 379, 25.15%	
	Mean (STD)	Mean (STD)	
Age (years)	42.81 (11.83)	45.92 (12.36)	< 0.001
Weight (kilograms)	70.33 (15.18)	72.09 (15.46)	0.051
Smoking intensity (number/day)	21.27 (11.48)	18.61 (12.47)	< 0.001
Smoking years	24.13 (11.73)	25.88 (12.86)	0.014
Family income level	7.94 (2.70)	8.15 (2.48)	0.173
	Number (%)	Number (%)	
Female	598 (53.0)	176 (46.4)	0.031
Black or other	161 (14.3)	34 (9.0)	0.01
Active life level			0.268
very active	514 (45.6)	156 (41.2)	
moderately active	515 (45.7)	183 (48.3)	
inactive	99 (8.8)	40 (10.6)	
Education level			0.01
8th grade or less	203 (18.0)	76 (20.1)	
high school dropout	252 (22.3)	72 (19.0)	
high school	471 (41.8)	144 (38.0)	
college dropout	91 (8.1)	26 (6.9)	
college or more	111 (9.8)	61 (16.1)	
Exercise situation			0.121
much exercise	233 (20.7)	60 (15.8)	
moderate exercise	473 (41.9)	168 (44.3)	
little or no exercise	422 (37.4)	151 (39.8)	
Ever use of weight loss medication 31 (2.7)		7 (1.8)	0.436

The continuous variable is presented as mean (standard deviance) and the difference between non-quitters and quitters is compared by t-test. The categorical variable is presented as counts (percentage) and the difference between non-quitters and quitters is compared by Chi-square test

To identify candidate models for ANN.MiPS estimator, we explored the association of smoking cessation with all potential risk factors by logistic regression, and explored the association of weight gain with all potential risk factors by linear regression. The covariates in model 1 and model 2 for both PS and OR models were identified at significant levels of 0.05 and 0.1, respectively. The covariates in PS model 1 and model 2 were (i) age, gender, race, smoking intensity, and smoking years; (ii) age, gender, race, smoking intensity, smoking years, education level, and exercise situation. The covariates in OR model 1 and model 2 were (i) age, weight at baseline, smoking intensity, education level, and active life level; (ii) age, weight at baseline, smoking intensity, education level, active life level, and family income level. We applied the single model-based IPW estimator, single model-based OR estimator, and our proposed ANN.MiPS estimator to estimate the ATE. The four numbers in the ANN.MiPS estimator, from left to right, represents if PS model 1, PS model 2, OR model 1, or OR model 2 is included in the estimator, where “1” indicates yes and “0” indicates no. For example, “ANN.MiPS-1010” represents that the PS model 1 and OR model 1 are included in the estimator. The standard error of estimation was estimated based on 500 resampled bootstrapping.

The estimation results in Table 5 indicated that all estimators suggested quitting smoking significantly increased participants' weight gain. Most of the estimated adjusted effects based on these estimators were greater than the estimated unadjusted effects of 2.4, which seems more precise and reliable. The point estimation and its bootstrap standard error for ATE of the ANN.MiPS estimator was stable under different model specifications.

**Table 5:** The NHEFS data analysis: estimated average treatment effect of quitting smoking on weight gain (not quitting smoking as reference)

Estimator	Estimates	BS-SE	95%-CI	*P*-value
Single model-based estimators				
IPW.model1	3.015	0.522	(1.992, 4.038)	< 0.001
IPW.model2	3.140	0.515	(2.131, 4.149)	< 0.001
IPW.ANN	2.404	0.560	(1.306, 3.502)	< 0.001
OR.model1	3.187	0.471	(2.264, 4.110)	< 0.001
OR.model2	3.254	0.477	(2.319, 4.189)	< 0.001
OR.ANN	3.392	0.825	(1.775, 5.009)	< 0.001
Artificial neural network-based MiPS estimator				
MiPS-1000	2.713	0.510	(1.713, 3.713)	< 0.001
MiPS-0100	2.871	0.510	(1.871, 3.871)	< 0.001
MiPS-0010	2.584	0.468	(1.667, 3.501)	< 0.001
MiPS-0001	2.221	0.476	(1.288, 3.154)	< 0.001
MiPS-1100	2.880	0.505	(1.890, 3.870)	< 0.001
MiPS-1010	2.764	0.508	(1.768, 3.760)	< 0.001
MiPS-1001	2.704	0.520	(1.685, 3.723)	< 0.001
MiPS-0110	2.834	0.513	(1.829, 3.839)	< 0.001
MiPS-0101	2.868	0.520	(1.849, 3.887)	< 0.001
MiPS-0011	2.606	0.468	(1.689, 3.523)	< 0.001
MiPS-1110	2.847	0.515	(1.838, 3.856)	< 0.001
MiPS-1101	2.890	0.528	(1.855, 3.925)	< 0.001
MiPS-1011	2.868	0.546	(1.798, 3.938)	< 0.001
MiPS-0111	2.854	0.536	(1.803, 3.905)	< 0.001
MiPS-1111	2.873	0.526	(1.842, 3.904)	< 0.001

*BS-SE* bootstrapping standard error based on 500 resamples, *95%-CI* 95% Wald confidence interval. The artificial neural network-based MiPS estimator which contains propensity score model and/or outcome regression model is denoted as “method-0000”, where each digit of the four numbers, from left to right, indicates if propensity score model 1, propensity score model 2, outcome regression model 1, outcome regression model 2 is included in the estimator (“1” indicates yes and “0” indicates no)

## Discussion

In this paper, we considered causal inference in observational studies where effects estimation was susceptible to confounding bias due to imbalanced covariates between groups. With reference to the Ker.DiPS estimator [19], we proposed the ANN.MiPS estimator to provide more chances for correcting the confounding bias. We evaluated the performance of our estimator under simulation scenarios with small ($n=300$) or large ($n=1000$) sample size, with treatment rate of 25% or 50%, and with covariates consisting of all continuous type or both continuous and discrete types. The results indicated the multiple robustness property of our estimator: the estimation bias is small if any model for PS or any model for OR is correctly specified. In addition to achieving multiply robust estimation for ATE, the proposed estimator showed a higher estimation efficiency than the kernel-based estimator when any model for PS or OR is correctly specified, especially when only the OR model is correctly specified.

One limitation of our approach is that the multiple candidate models for PS $\{{\pi }^{k}\left(\mathbf{X};{\boldsymbol{\alpha }}^{k}\right)={g}_{\pi }\left({\alpha }_{0}^{k}+{\boldsymbol{\alpha }}_{1}^{kT}\mathbf{X}\right),k=1,\dots ,K\}$ and the multiple candidate models for OR $\left\{{\mu }^{l}\left(\mathbf{X},A;{{\varvec{\beta}}}^{l}\right)={g}_{\mu }\left({\beta }_{1}^{l}+{{\varvec{\beta}}}_{1}^{lT}\mathbf{X}+{\beta }_{2}^{l}A\right),l=1,\dots ,L\right\}$ need to be parametric, since the MiPS is defined as $\pi \left(\mathbf{X};{\boldsymbol{\alpha }}_{1}^{1},...,{\boldsymbol{\alpha }}_{1}^{K},{{\varvec{\beta}}}_{1}^{1},...,{{\varvec{\beta}}}_{1}^{L}\right)=E\left[A |{\boldsymbol{\alpha }}_{1}^{1T}\mathbf{X},...{\boldsymbol{\alpha }}_{1}^{KT}\mathbf{X},{{\varvec{\beta}}}_{1}^{1T}\mathbf{X},...,{{\varvec{\beta}}}_{1}^{LT}\mathbf{X}\right]$, in which we need to conduct nonparametric regression of $A$ on ${\widehat{\boldsymbol{\alpha }}}_{1}^{1\mathrm{T}}\mathbf{X}$,…, ${\widehat{\boldsymbol{\alpha }}}_{1}^{K\mathrm{T}}\mathbf{X}$ and ${\widehat{{\varvec{\beta}}}}_{1}^{1\mathrm{T}}\mathbf{X}$,…, ${\widehat{{\varvec{\beta}}}}_{1}^{L\mathrm{T}}\mathbf{X}$. Therefore, the nonparametric models, such as the kernel function, ANN, and random forest are not suitable as candidate models for the MiPS estimator because the coefficients of covariates cannot be obtained. When the candidate models are constructed by nonparametric models, some other multiply robust approaches may be adopted to integrate the information from multiple candidate models, such as the regression-based estimator under least square’s framework [40], the estimator based on empirical likelihood weighting [20], and the estimator based on model mixture procedures [41]. At this point, double/debiased machine learning approach may be extended to multiple/debiased machine learning for obtaining valid inference about ATE [42].

Although the performance of ANN.MiPS estimator remains stable when specifying eight candidate models, an excessive number of models can impose a heavy computational burden. Therefore, we recommend carefully constructing a comprehensive set of reasonable but less similar candidate models to control the model number in practical applications, using both subject knowledge and reliable data-driven tools, such as causality diagrams [43], variable selection techniques [44], and covariate balancing diagnostics [45].

Finally, we give some intuitive discussions about the theoretical properties of the proposed estimator. Referring to proof Chen et al. [19], ${\widehat{\Delta }}_{MiPS}^{ANN}$ is consistent for

$${\overline{\Delta } }_{MiPS}^{ANN}=\frac{E\left\{\frac{{A}_{i}{Y}_{i}}{{\overline{\pi }}^{ANN}\left({\mathbf{X}}_{i};{\overline{\boldsymbol{\alpha }} }_{1}^{1},...,{\overline{\boldsymbol{\alpha }} }_{1}^{K},{\overline{{\varvec{\beta}}} }_{1}^{1},...,{\overline{{\varvec{\beta}}} }_{1}^{L}\right)}\right\}}{E\left\{\frac{{A}_{i}}{{\overline{\pi }}^{ANN}\left({\mathbf{X}}_{i};{\overline{\boldsymbol{\alpha }} }_{1}^{1},...,{\overline{\boldsymbol{\alpha }} }_{1}^{K},{\overline{{\varvec{\beta}}} }_{1}^{1},...,{\overline{{\varvec{\beta}}} }_{1}^{L}\right)}\right\}}-\frac{E\left\{\frac{\left(1-{A}_{i}\right){Y}_{i}}{\left[1-{\overline{\pi }}^{ANN}\left({\mathbf{X}}_{i};{\overline{\boldsymbol{\alpha }} }_{1}^{1},...,{\overline{\boldsymbol{\alpha }} }_{1}^{K},{\overline{{\varvec{\beta}}} }_{1}^{1},...,{\overline{{\varvec{\beta}}} }_{1}^{L}\right)\right]}\right\}}{E\left\{\frac{\left(1-{A}_{i}\right)}{\left[1-{\overline{\pi }}^{ANN}\left({\mathbf{X}}_{i};{\overline{\boldsymbol{\alpha }} }_{1}^{1},...,{\overline{\boldsymbol{\alpha }} }_{1}^{K},{\overline{{\varvec{\beta}}} }_{1}^{1},...,{\overline{{\varvec{\beta}}} }_{1}^{L}\right)\right]}\right\}}$$

where ${\widehat{\boldsymbol{\alpha }}}_{1}^{1},...,{\widehat{\boldsymbol{\alpha }}}_{1}^{K},{\widehat{{\varvec{\beta}}}}_{1}^{1},...,{\widehat{{\varvec{\beta}}}}_{1}^{L}$ converge to ${\overline{\boldsymbol{\alpha }} }_{1}^{1},...,{\overline{\boldsymbol{\alpha }} }_{1}^{K},{\overline{{\varvec{\beta}}} }_{1}^{1},...,{\overline{{\varvec{\beta}}} }_{1}^{L}$, ${\widehat{\pi }}^{ANN}\left(\bullet \right)$ converges to ${\overline{\pi }}^{ANN}\left(\bullet \right)$. According to some theoretical results on ANN, under certain conditions, ${\overline{\pi }}^{ANN}\left(\mathbf{X};{\overline{\boldsymbol{\alpha }} }_{1}^{1},...,{\overline{\boldsymbol{\alpha }} }_{1}^{K},{\overline{{\varvec{\beta}}} }_{1}^{1},...,{\overline{{\varvec{\beta}}} }_{1}^{L}\right)=\pi \left(\mathbf{X};{\overline{\boldsymbol{\alpha }} }_{1}^{1},...,{\overline{\boldsymbol{\alpha }} }_{1}^{K},{\overline{{\varvec{\beta}}} }_{1}^{1},...,{\overline{{\varvec{\beta}}} }_{1}^{L}\right)$. At this time, when one of candidate models for PS $\{{\pi }^{k}\left(\mathbf{X};{\boldsymbol{\alpha }}^{k}\right)={g}_{\pi }\left({\alpha }_{0}^{k}+{\boldsymbol{\alpha }}_{1}^{kT}\mathbf{X}\right),k=1,\dots ,K\}$ is correctly specified, $\pi \left(\mathbf{X};{\overline{\boldsymbol{\alpha }} }_{1}^{1},...,{\overline{\boldsymbol{\alpha }} }_{1}^{K},{\overline{{\varvec{\beta}}} }_{1}^{1},...,{\overline{{\varvec{\beta}}} }_{1}^{L}\right)=\pi \left(\mathbf{X}\right)$, ${\overline{\Delta } }_{MiPS}^{ANN}=\Delta$. On the other hand, when one of candidate models for OR $\left\{{\mu }_{A}^{l}\left(\mathbf{X};{{\varvec{\beta}}}^{l}\right)={g}_{\mu }\left({\beta }_{1}^{l}+{{\varvec{\beta}}}_{1}^{lT}\mathbf{X}+{\beta }_{2}^{l}A\right),l=1,\dots ,L\right\}$ is correctly specified, $E\left[Y |{\overline{\boldsymbol{\alpha }} }_{1}^{1T}\mathbf{X},...{\overline{\boldsymbol{\alpha }} }_{1}^{KT}\mathbf{X},{\overline{{\varvec{\beta}}} }_{1}^{1T}\mathbf{X},...,{\overline{{\varvec{\beta}}} }_{1}^{LT}\mathbf{X},A \right]={\mu }_{A}\left(\mathbf{X}\right)$, ${\overline{\Delta } }_{MiPS}^{ANN}=\Delta$. As for the asymptotic distribution of proposed estimator, the variability of ${\widehat{\Delta }}_{MiPS}^{ANN}$ mainly comes from: (1) the estimated values ${\widehat{\boldsymbol{\alpha }}}_{1}^{1}$,…, ${\widehat{\boldsymbol{\alpha }}}_{1}^{K}$ of multiple PS models and the estimated values ${\widehat{{\varvec{\beta}}}}_{1}^{1}$,…, ${\widehat{{\varvec{\beta}}}}_{1}^{L}$ of multiple OR models, (2) the estimated nonparametric function ${\widehat{\pi }}^{ANN}\left(\bullet \right)$ using ANN. For the first variation, if the parameters are estimated by maximum likelihood, the asymptotic normality of the estimators has been obtained by White [46]. For the second variation, the error bound and convergence rate have been discussed in some theoretical research [29, 47]. It will be our future research topic to give and prove the theoretical properties of ${\widehat{\Delta }}_{MiPS}^{ANN}$ estimator strictly and systematically.

## Conclusions

IN this study, we proposed the ANN.MiPS estimator to correct confounding bias when using the observational data to estimate the ATE. The proposed estimator allowed multiple candidate models for PS and OR, and guaranteed the estimated integrated PS is between 0 and 1. The multiple robustness property of our estimator was illustrated through simulation studies. Extra efficiency was gained compared to the kernel function-based estimator. The proposed estimator provided a new choice for multiply robust estimation of ATE in observational studies.

## Supplementary Information

**Additional file 1: Fig. S1**. The distribution of the estimated average treatment effect for kernel-based MiPS estimator and artificial neural network-based MiPS estimator in 1000 simulated data sets. The range of the y-axis is restricted from -1.4 to 0.6 given that the kernel-based MiPS estimator yields highly biased estimation under some model specifications. The dashed line denotes the true average treatment effect. **Table S1.** Estimation results for scenario with both continuous and discrete covariates under 50% treated based on 1000 replications. **Table S2.** Estimation results of multi-index propensity score estimator incorporating extra incorrect models under scenario with both continuous and discrete covariates. **Table S3.** Sensitivity analysis of ANN.MiPS estimator with different tuning parameters selection for ANN under scenario of all continuous covariates and 50% treated.

## Data Availability

The simulated data can be simulated from the example code in the attachment. The real-world data used can be accessed from https://wwwn.cdc.gov/nchs/nhanes/nhefs/default.aspx/.

## Abbreviations

ATE: Average treatment effect
IPW: Inverse probability weighting
PS: Propensity score
OR: Outcome regression
AIPW: Augment inverse probability weighting
TMLE: Target maximum likelihood estimator
DiPS: Double-index propensity score
Ker.DiPS: Kernel function-based double-index propensity score
MiPS: Multi-index propensity score
ANN: Artificial neural network
ANN.MiPS: Artificial neural network-based multi-index propensity score
Ker.MiPS: Kernel function-based multi-index propensity score
RMSE: Root mean square error
MC-SE: Monte Carlo standard error
BS-SE: Bootstrapping standard error
95CI-Cov: 95% Confidence interval coverage rate
NHEFS: Nutrition Examination Survey Data | Epidemiologic Follow-up Study
