# Supplementary material for: Estimation of average treatment effect based on a multi-index propensity score
Source: BMC Med Res Methodol. 2022 Dec 28;22:337. doi: 10.1186/s12874-022-01822-3 (PMC9795597; doi:10.1186/s12874-022-01822-3)
Supplement: Supplementary file 1 — Additional file 1: Fig. S1. The distribution of the estimated average treatment effect for kernel-based MiPS estimator and artificial neural network-based MiPS estimator in 1000 simulated data sets. The range of the y-axis is restricted from -1.4 to 0.6 given that the kernel-based MiPS estimator yields highly biased estimation under some model specifications. The dashed line denotes the true average treatment effect. Table S1. Estimation results for scenario with both continuous and discrete covariates under 50% treated based on 1000 replications. Table S2. Estimation results of multi-index propensity score estimator incorporating extra incorrect models under scenario with both continuous and discrete covariates. Table S3. Sensitivity analysis of ANN.MiPS estimator with different tuning parameters selection for ANN under scenario of all continuous covariates and 50% treated. [file 12874_2022_1822_MOESM1_ESM.zip › Supplementary Materials/Supplementary Document.docx]

**SIMULATION SETUP FOR THE SCENARIO WITH BOTH CONTINUOUS AND DISCRETE COVARIATES**

The simulation structure was same as the scenario with all continuous covariates described in the main text. Four covariates $(X_{1}, X_{2}, X_{3}, X_{4})$ were associated with both treatment and outcome. Three covariates $(X_{5}, X_{6}, X_{7})$ were treatment predictors that were only associated with treatment assignment. Three covariates ${(X}_{8}, X_{9}, X_{10})$ were outcome predictors that were only associated with outcome.

The type and distribution of covariates were different from that of main text. Four covariates ${(X}_{1}, X_{4},X_{7}, X_{10})$ were generated from the standard normal distribution. One covariate $X_{5}$ was generated from the normal distribution with mean 0.5 and standard deviation 3. One covariate $X_{9}$ was generated from the normal distribution with mean 2 and standard deviation 3. Three covariates $(X_{2}, X_{6},X_{8})$were generated from the binomial distribution with probability of 0.5, 0.5, and 0.7, respectively. One covariate $X_{3}$ was generated from the Poisson distribution with mean 1.5.

All correlation coefficients between covariates were set to 0. The binary treatment indicator $A$ was generated from a Bernoulli distribution according to the following propensity score

$$\mathrm{logit} \left[ \pi\left( \mathbf{X};\alpha\right) \right]=\alpha_{0}+0.16X_{1}-0.05X_{2}+0.12X_{3}-$$

$$0.1X_{4}-0.16X_{5}-0.1X_{6}+0.15X_{7}$$

$\alpha_{0}$ was set to be 0 to make approximately 50% subjects entering the treatment group. The continuous outcome $Y$ was generated from a linear combination of treatment $A$ and covariates $\mathbf{X}$

$$Y=-3.85-0.4A-0.8X_{1}-0.36X_{2}-0.73X_{3}-$$

$$0.2X_{4}+0.71X_{8}-0.19X_{9}+0.26X_{10}+\varepsilon,$$

where $\varepsilon$ had an independent $N(0,1)$ distribution. The true ATE was $\Delta=E\left( Y^{1} \right)-E\left( Y^{0} \right)=-0.4$.

**Table S1.** Estimation results for scenario with both continuous and discrete covariates under 50% treated based on 1000 replications.

|  | n=300 | | | | |  | n=1000 | | | | | |
| --- | --- | --- | --- | --- | --- | --- | --- | --- | --- | --- | --- | --- |
| Estimator | BIAS(%) | RMSE | MC-SE | BS-SE | CI-Cov(%) |  | BIAS(%) | RMSE | MC-SE | BS-SE | CI-Cov(%) | |
| Single model-based estimator | | | | | | | | | | | |  |
| IPW.correct | -1.279 | 0.153 | 0.153 | 0.152 | 94.8 |  | -1.119 | 0.086 | 0.086 | 0.081 | 93.8 | |
| IPW.incorrect | 7.432 | 0.156 | 0.153 | 0.155 | 94.4 |  | 8.008 | 0.090 | 0.084 | 0.082 | 91.6 | |
| IPW.ANN | -1.717 | 0.162 | 0.162 | 0.439 | 100.0 |  | 0.357 | 0.088 | 0.088 | 0.115 | 99.2 | |
| OR.correct | -2.471 | 0.114 | 0.114 | 0.117 | 96.0 |  | -0.933 | 0.066 | 0.066 | 0.063 | 93.2 | |
| OR.incorrect | 7.130 | 0.139 | 0.136 | 0.138 | 95.0 |  | 8.770 | 0.083 | 0.075 | 0.075 | 91.4 | |
| OR.ANN | -6.353 | 0.138 | 0.136 | 0.168 | 98.8 |  | -4.476 | 0.081 | 0.079 | 0.085 | 96.4 | |
| Doubly robust estimator | | | | | | | | | | | |  |
| AIPW-1010 | 0.285 | 0.116 | 0.116 | 0.122 | 95.6 |  | 0.361 | 0.069 | 0.069 | 0.066 | 93.4 | |
| AIPW-1001 | 1.053 | 0.141 | 0.141 | 0.142 | 94.4 |  | 0.247 | 0.079 | 0.079 | 0.076 | 94.2 | |
| AIPW-0110 | 0.988 | 0.116 | 0.115 | 0.119 | 95.8 |  | 0.355 | 0.066 | 0.066 | 0.064 | 93.4 | |
| AIPW-0101 | -2.643 | 0.140 | 0.138 | 0.143 | 94.8 |  | -3.500 | 0.083 | 0.076 | 0.075 | 91.8 | |
| TMLE-1010 | 1.118 | 0.121 | 0.121 | 0.123 | 94.8 |  | 0.341 | 0.069 | 0.069 | 0.066 | 93.0 | |
| TMLE-1001 | 1.118 | 0.121 | 0.121 | 0.123 | 94.8 |  | 0.341 | 0.069 | 0.069 | 0.066 | 93.0 | |
| TMLE-0110 | 1.118 | 0.121 | 0.121 | 0.123 | 94.8 |  | 0.341 | 0.069 | 0.069 | 0.066 | 93.0 | |
| TMLE-0101 | -2.605 | 0.140 | 0.138 | 0.140 | 95.2 |  | -3.463 | 0.083 | 0.075 | 0.075 | 91.8 | |
| Kernel regression-based MiPS estimator | | | | | | | | | | | |  |
| MiPS-1000 | 1.388 | 0.156 | 0.156 | 0.193 | 96.4 |  | 0.136 | 0.086 | 0.086 | 0.160 | 97.2 | |
| MiPS-0100 | 7.944 | 0.320 | 0.319 | 0.303 | 97.8 |  | 8.262 | 0.362 | 0.361 | 0.354 | 96.8 | |
| MiPS-0010 | 0.414 | 0.358 | 0.359 | 0.267 | 96.6 |  | -3.659 | 0.314 | 0.314 | 0.375 | 97.0 | |
| MiPS-0001 | 10.986 | 0.263 | 0.260 | 0.275 | 96.2 |  | 7.218 | 0.427 | 0.426 | 0.288 | 95.4 | |
| MiPS-1100 | 6.450 | 0.176 | 0.174 | 0.249 | 96.6 |  | 9.093 | 0.408 | 0.407 | 0.507 | 96.4 | |
| MiPS-1010 | 9.209 | 0.137 | 0.132 | 0.144 | 95.4 |  | 6.279 | 0.078 | 0.074 | 0.141 | 95.0 | |
| MiPS-1001 | 11.273 | 0.152 | 0.145 | 0.166 | 94.8 |  | 6.805 | 0.133 | 0.130 | 0.239 | 96.4 | |
| MiPS-0110 | 8.753 | 0.135 | 0.130 | 0.154 | 94.6 |  | 4.230 | 0.199 | 0.199 | 0.260 | 97.8 | |
| MiPS-0101 | 15.883 | 0.160 | 0.147 | 0.194 | 95.2 |  | 12.670 | 0.351 | 0.347 | 0.397 | 97.4 | |
| MiPS-0011 | 8.901 | 0.384 | 0.383 | 0.417 | 96.2 |  | 6.605 | 0.763 | 0.763 | 0.797 | 95.0 | |
| MiPS-1110 | 17.268 | 0.160 | 0.145 | 0.144 | 92.0 |  | 15.059 | 0.099 | 0.078 | 0.088 | 89.4 | |
| MiPS-1101 | 17.940 | 0.171 | 0.155 | 0.151 | 91.0 |  | 14.939 | 0.113 | 0.096 | 0.116 | 91.6 | |
| MiPS-1011 | 15.354 | 0.153 | 0.140 | 0.140 | 92.6 |  | 11.137 | 0.127 | 0.119 | 0.156 | 94.2 | |
| MiPS-0111 | 14.826 | 0.151 | 0.139 | 0.142 | 93.6 |  | 11.077 | 0.149 | 0.143 | 0.224 | 97.0 | |
| MiPS-1111 | 22.291 | 0.179 | 0.155 | 0.152 | 90.0 |  | 20.229 | 0.116 | 0.083 | 0.080 | 81.0 | |
| Artificial neural network-based MiPS estimator | | | | | | | | | | | |  |
| MiPS-1000 | 0.701 | 0.153 | 0.153 | 0.151 | 94.8 |  | -0.221 | 0.085 | 0.085 | 0.080 | 93.8 | |
| MiPS-0100 | 16.618 | 0.180 | 0.167 | 0.155 | 91.2 |  | 11.271 | 0.097 | 0.086 | 0.083 | 90.6 | |
| MiPS-0010 | 0.122 | 0.119 | 0.119 | 0.122 | 95.6 |  | -0.404 | 0.067 | 0.067 | 0.064 | 92.8 | |
| MiPS-0001 | 9.994 | 0.146 | 0.140 | 0.141 | 94.6 |  | 8.989 | 0.082 | 0.074 | 0.074 | 91.2 | |
| MiPS-1100 | -4.272 | 0.156 | 0.155 | 0.155 | 94.6 |  | -3.746 | 0.090 | 0.088 | 0.083 | 93.2 | |
| MiPS-1010 | -1.221 | 0.130 | 0.130 | 0.137 | 96.4 |  | 0.303 | 0.072 | 0.072 | 0.069 | 93.6 | |
| MiPS-1001 | -0.967 | 0.146 | 0.146 | 0.150 | 95.8 |  | 0.105 | 0.079 | 0.080 | 0.077 | 94.2 | |
| MiPS-0110 | -3.122 | 0.123 | 0.123 | 0.128 | 96.2 |  | -1.956 | 0.068 | 0.067 | 0.065 | 94.0 | |
| MiPS-0101 | 5.701 | 0.144 | 0.142 | 0.145 | 95.4 |  | 6.824 | 0.079 | 0.074 | 0.075 | 92.4 | |
| MiPS-0011 | -0.068 | 0.118 | 0.119 | 0.123 | 96.4 |  | -0.037 | 0.067 | 0.067 | 0.065 | 93.0 | |
| MiPS-1110 | -1.907 | 0.133 | 0.133 | 0.140 | 96.6 |  | 0.086 | 0.072 | 0.072 | 0.069 | 93.6 | |
| MiPS-1101 | -1.506 | 0.147 | 0.147 | 0.153 | 96.0 |  | 0.041 | 0.079 | 0.079 | 0.077 | 94.6 | |
| MiPS-1011 | -0.921 | 0.130 | 0.130 | 0.138 | 96.4 |  | 0.489 | 0.072 | 0.072 | 0.070 | 93.2 | |
| MiPS-0111 | -1.683 | 0.121 | 0.121 | 0.129 | 96.2 |  | -1.097 | 0.067 | 0.067 | 0.066 | 94.6 | |
| MiPS-1111 | -1.033 | 0.132 | 0.132 | 0.141 | 96.6 |  | 0.301 | 0.072 | 0.072 | 0.070 | 94.0 | |

BIAS: bias. RMSE: root mean square error. MC-SE: Monte Carlo standard error. BS-SE: bootstrapping standard error. CI-Cov, coverage rate of 95% Wald confidence interval.

AIPW: augmented inverse probability weighting. TMLE: target maximum likelihood estimator. IPW.ANN: artificial neural network-based inverse probability weighting estimator. OR.ANN: artificial neural network-based outcome regression estimator. MiPS: multi-index propensity score. IPW: inverse probability weighting. OR: outcome regression.

The estimator which contains correct and/or incorrect models for propensity score and/or outcome regression is denoted as “method-0000”, where each digit of the four numbers, from left to right, indicates if $\pi^{1}\left( \boldsymbol{X};\boldsymbol{\alpha}^{1} \right)$, $\pi^{2}\left( \boldsymbol{X};\boldsymbol{\alpha}^{2} \right)$, ${\mu_{A}}^{1}\left( \boldsymbol{X};\boldsymbol{\beta}^{1} \right)$ or ${\mu_{A}}^{2}\left( \boldsymbol{X};\boldsymbol{\beta}^{2} \right)$ is included in the estimator (“1” indicates yes and “0” indicates no).

**Table S2.** Estimation results of multi-index propensity score estimator incorporating extra incorrect models under scenario with both continuous and discrete covariates.

|  | n=300 | | | | |  | n=1000 | | | | |
| --- | --- | --- | --- | --- | --- | --- | --- | --- | --- | --- | --- |
| Estimator | BIAS(%) | RMSE | MC-SE | BS-SE | CI-Cov(%) |  | BIAS(%) | RMSE | MC-SE | BS-SE | CI-Cov(%) |
| Under 25% treated | | | | | | | | | | | |
| Kernel regression-based MiPS estimator | | | | | | | | | | | |
| MiPS-1111-2PS | 27.931 | 0.205 | 0.172 | 0.168 | 87.2 |  | 27.258 | 0.142 | 0.091 | 0.090 | 77.2 |
| MiPS-1111-2OR | 27.597 | 0.203 | 0.170 | 0.166 | 87.8 |  | 26.331 | 0.139 | 0.090 | 0.089 | 77 |
| MiPS-1111-2PS2OR | 29.697 | 0.214 | 0.178 | 0.173 | 86.8 |  | 29.235 | 0.150 | 0.094 | 0.094 | 75.4 |
| Artificial neural network-based MiPS estimator | | | | | | | | | | | |
| MiPS-1111-2PS | -1.367 | 0.133 | 0.133 | 0.142 | 96.6 |  | 0.249 | 0.072 | 0.072 | 0.070 | 93.6 |
| MiPS-1111-2OR | -0.875 | 0.133 | 0.133 | 0.143 | 96.0 |  | 0.429 | 0.073 | 0.073 | 0.071 | 94.2 |
| MiPS-1111-2PS2OR | -1.080 | 0.132 | 0.132 | 0.144 | 97.4 |  | 0.379 | 0.073 | 0.073 | 0.071 | 94.4 |

BIAS: bias. RMSE: root mean square error. MC-SE: Monte Carlo standard error. BS-SE: bootstrapping standard error. CI-Cov, coverage rate of 95% Wald confidence interval. MiPS: multi-index propensity score.

MiPS-1111-2PS indicates the estimator with two additional incorrect propensity score models on the basis of MiPS-1111 estimator.

MiPS-1111-2OR indicates the estimator with two additional incorrect outcome regression models on the basis of MiPS-1111 estimator.

MiPS-1111-2PS2OR indicates the estimator with two additional two incorrect propensity score and 2 incorrect outcome regression models on the basis of MiPS-1111 estimator.

**Table S3.** Sensitivity analysis of ANN.MiPS estimator with different tuning parameters selection for ANN under scenario of all continuous covariates and 50% treated.

|  | n=300 | | | | |  | n=1000 | | | | |
| --- | --- | --- | --- | --- | --- | --- | --- | --- | --- | --- | --- |
| Estimator | BIAS(%) | RMSE | MC-SE | BS-SE | CI-Cov(%) |  | BIAS(%) | RMSE | MC-SE | BS-SE | CI-Cov(%) |
| 1 hidden layer consisting of 5 neurons, momentum 0.3, learning rate 0.003 | | | | | | | | | | | |
| MiPS-1111-2PS | 0.075 | 0.128 | 0.128 | 0.142 | 97.3 |  | 0.425 | 0.069 | 0.069 | 0.069 | 95.3 |
| MiPS-1111-2OR | -0.105 | 0.125 | 0.126 | 0.145 | 96.8 |  | 0.524 | 0.071 | 0.071 | 0.073 | 96.0 |
| MiPS-1111-2PS2OR | 0.044 | 0.125 | 0.125 | 0.147 | 97.3 |  | 0.429 | 0.070 | 0.070 | 0.072 | 96.7 |
| 1 hidden layer consisting of 5 neurons, momentum 0.7, learning rate 0.003 | | | | | | | | | | | |
| MiPS-1111-2PS | -0.747 | 0.126 | 0.126 | 0.157 | 98.0 |  | 0.563 | 0.074 | 0.074 | 0.084 | 98.3 |
| MiPS-1111-2OR | -0.164 | 0.126 | 0.126 | 0.176 | 97.4 |  | 0.809 | 0.080 | 0.080 | 0.099 | 99.3 |
| MiPS-1111-2PS2OR | -0.490 | 0.127 | 0.128 | 0.189 | 98.7 |  | 0.481 | 0.079 | 0.079 | 0.102 | 99.0 |
| 2 hidden layers consisting of 4,4 neurons, momentum 0.3, learning rate 0.003 | | | | | | | | | | | |
| MiPS-1111-2PS | 0.388 | 0.131 | 0.132 | 0.144 | 96.3 |  | 0.501 | 0.070 | 0.070 | 0.070 | 95.3 |
| MiPS-1111-2OR | 0.331 | 0.129 | 0.129 | 0.146 | 97.0 |  | 0.701 | 0.071 | 0.071 | 0.075 | 97.0 |
| MiPS-1111-2PS2OR | 0.123 | 0.127 | 0.127 | 0.149 | 97.2 |  | 0.284 | 0.071 | 0.071 | 0.075 | 96.3 |
| 2 hidden layers consisting of 4,4 neurons, momentum 0.7, learning rate 0.003 | | | | | | | | | | | |
| MiPS-1111-2PS | -0.261 | 0.130 | 0.130 | 0.168 | 98.0 |  | 0.724 | 0.073 | 0.073 | 0.085 | 98.7 |
| MiPS-1111-2OR | -0.004 | 0.128 | 0.128 | 0.195 | 99.7 |  | 0.596 | 0.077 | 0.077 | 0.099 | 100.0 |
| MiPS-1111-2PS2OR | -0.138 | 0.128 | 0.128 | 0.227 | 100.0 |  | 0.361 | 0.077 | 0.077 | 0.103 | 99.7 |
| 3 hidden layers consisting of 3,3,3 neurons, momentum 0.3, learning rate 0.003 | | | | | | | | | | | |
| MiPS-1111-2PS | -0.779 | 0.141 | 0.141 | 0.155 | 97.4 |  | 0.161 | 0.070 | 0.070 | 0.073 | 95.3 |
| MiPS-1111-2OR | -0.902 | 0.139 | 0.140 | 0.156 | 97.0 |  | 0.664 | 0.071 | 0.071 | 0.077 | 96.7 |
| MiPS-1111-2PS2OR | 0.457 | 0.138 | 0.138 | 0.158 | 97.0 |  | 0.188 | 0.073 | 0.073 | 0.077 | 96.0 |
| 3 hidden layers consisting of 3,3,3 neurons, momentum 0.7, learning rate 0.003 | | | | | | | | | | | |
| MiPS-1111-2PS | -0.322 | 0.133 | 0.133 | 0.165 | 97.3 |  | 0.212 | 0.074 | 0.074 | 0.083 | 97.7 |
| MiPS-1111-2OR | -0.198 | 0.135 | 0.135 | 0.177 | 98.3 |  | 0.191 | 0.077 | 0.077 | 0.094 | 98.3 |
| MiPS-1111-2PS2OR | -0.148 | 0.135 | 0.135 | 0.190 | 99.3 |  | 0.005 | 0.078 | 0.078 | 0.096 | 99.7 |

BIAS: bias. RMSE: root mean square error. MC-SE: Monte Carlo standard error. BS-SE: bootstrapping standard error. CI-Cov, coverage rate of 95% Wald confidence interval. MiPS: multi-index propensity score.

MiPS-1111-2PS indicates the estimator with two additional incorrect propensity score models on the basis of MiPS-1111 estimator.

MiPS-1111-2OR indicates the estimator with two additional incorrect outcome regression models on the basis of MiPS-1111 estimator.

MiPS-1111-2PS2OR indicates the estimator with two additional two incorrect propensity score and 2 incorrect outcome regression models on the basis of MiPS-1111 estimator.


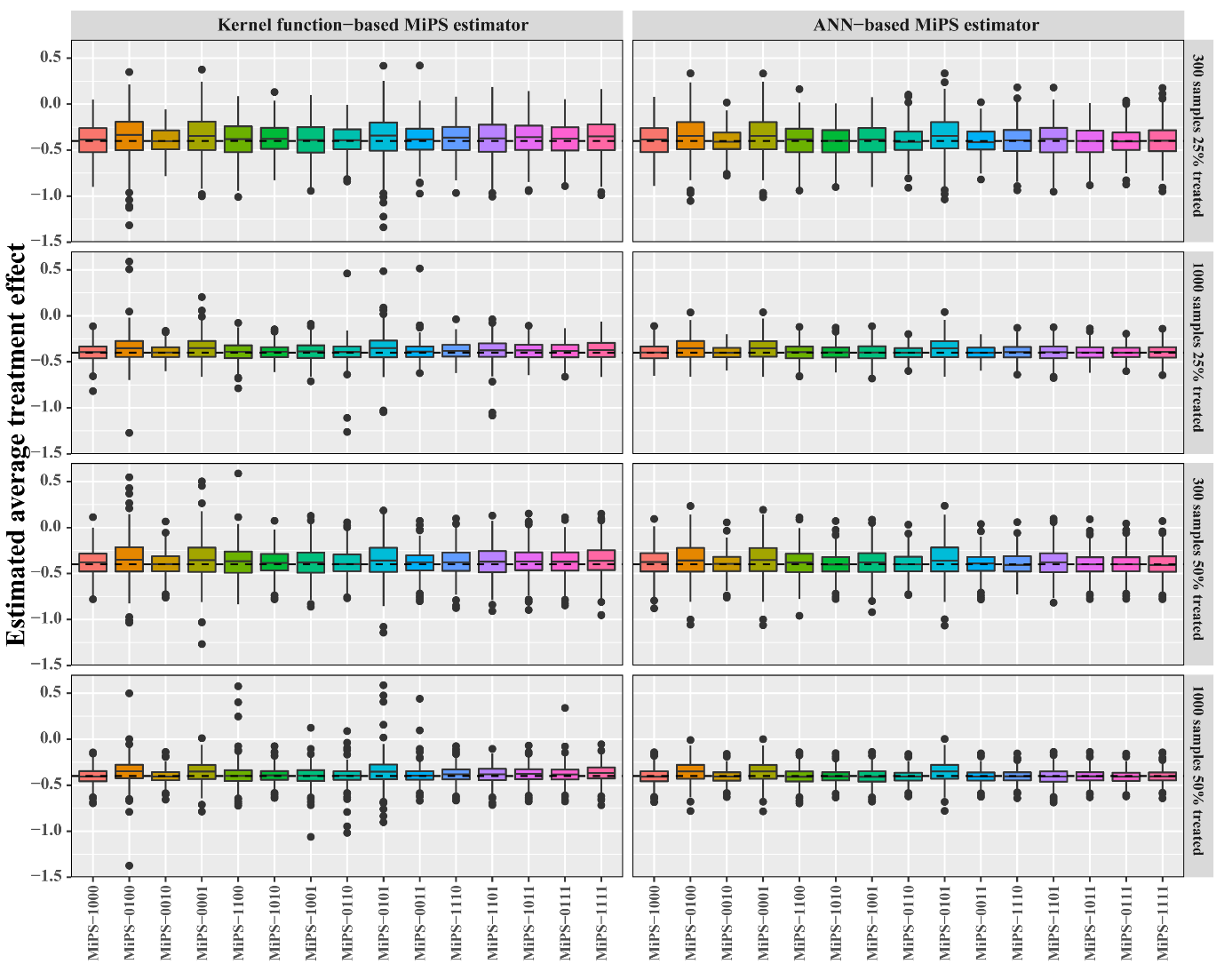


**Fig. S1** The distribution of the estimated average treatment effect for kernel-based MiPS estimator and artificial neural network-based MiPS estimator in 1000 simulated data sets.

The range of the y-axis is restricted from -1.4 to 0.6 given that the kernel-based MiPS estimator yields highly biased estimation under some model specifications. The dashed line denotes the true average treatment effect.
